# Examining sex disparity in the association of waist circumference, waist-hip ratio and BMI with hypertension among older adults in India

**DOI:** 10.1038/s41598-022-17518-z

**Published:** 2022-07-30

**Authors:** T. Muhammad, Ronak Paul, Rashmi Rashmi, Shobhit Srivastava

**Affiliations:** International Institute for Population Sciences, Mumbai, 400088 India

**Keywords:** Geriatrics, Public health

## Abstract

Hypertension is a public health issue touted as a “silent killer” worldwide. The present study aimed to explore the sex differential in the association of anthropometric measures including body mass index, waist circumference, and waist-hip ratio with hypertension among older adults in India. The study used data from the Longitudinal Aging Study in India (LASI) conducted during 2017–18. The sample contains 15,098 males and 16,366 females aged 60 years and above. Descriptive statistics (percentages) along with bivariate analysis were presented. Multivariable binary logistic regression analyses were used to examine the associations between the outcome variable (hypertension) and putative risk or protective factors. About 33.9% of males and 38.2% of females aged 60 years and above suffered from hypertension. After adjusting for the socioeconomic, demographic and health-behavioral factors, the odds of hypertension were 1.37 times (CI: 1.27–1.47), significantly higher among older adults who were obese or overweight than those with no overweight/obese condition. Older adults with high-risk waist circumference and waist-hip ratio had 1.16 times (CI: 1.08–1.25) and 1.42 times (CI: 1.32–1.51) higher odds of suffering from hypertension, respectively compared to their counterparts with no high-risk waist circumference or waist-hip ratio. The interaction effects showed that older females with overweight/obesity [OR: 0.84; CI: 0.61–0.74], high-risk waist circumference [OR: 0.89; CI: 0.78–0.99], and high-risk waist-hip ratio [OR: 0.90; CI: 0.83–0.97] had a lower chance of suffering from hypertension than their male counterparts with the similar anthropometric status. The findings suggested a larger magnitude of the association between obesity, high-risk waist circumference, high-risk waist-hip ratio and prevalent hypertension among older males than females. The study also highlights the importance of measuring obesity and central adiposity in older individuals and using such measures as screening tools for timely identification of hypertension.

## Introduction

Hypertension is a public health issue touted as a “silent killer” worldwide. It disproportionately affects the people of low- and middle-income countries with weak healthcare systems^1,2^. Globally, cardiovascular diseases (CVD) have emerged as the primary cause of death and disability, and the highest burden of CVD is attributable to hypertension^3^. In 2017, high systolic blood pressure was the leading cause for 10.4 million deaths worldwide^4^. With the shift in age dimensions worldwide, the threat of increasing cases of chronic conditions hiked, especially among the higher age groups. As a developing country, India is also observing demographic transition with a projected 20% increase in 60 years and above population by 2050. Besides being prevalent among older adults, a substantial sex differential has also been noticed in hypertension. For instance, in India, 3 out of 10 males and 4 out of 10 females were diagnosed with hypertension in 2018^5^. Evidence from the National Family Health Survey 2015–16 showed a higher prevalence of hypertension among men; however, at the age of 40 years and above, females were at higher risk than their male counterparts^6^.

Globally, various behavioural, socioeconomic, demographic and genetic factors have been linked with the occurrence of hypertension^1^. In the Indian scenario, existing literature had identified increasing age, obesity, smoking, diabetes and extra salt intake as common risk factors of hypertension^7–9^. Several studies have provided evidence on the mechanisms of obesity-induced hypertension^10–12^. Being a behavioural risk factor of hypertension, obesity has seen a threefold increase since 1975^13^. In India, 29% and 38% of obese male and female between 15 and 49 years were hypertensive^14^. Subsequently, one study based on the Indian city of Mangalore also showed that obese adults aged 20 years and above were three times more likely to experience hypertension. In the Indian context, several systematic reviews have concluded that obese adults have a greater risk of being hypertensive^7,8,15^. A series of cross-sectional studies (1995–2015) in the Indian city of Jaipur had concluded that obesity measured in terms of increased Body mass index (BMI), waist-circumference and waist-hip ratio was significantly associated with the increasing risk of hypertension among older adults^16,17^. BMI is the most popular anthropometric indicator for measuring obesity among adults. Extant research has also used waist-circumference, hip circumference and waist-hip ratio as measures of central-obesity, which have systematic advantages over BMI^18^. A primary study covering the slums of the Indian city of Tirupati had reported that higher BMI increased the risk of self-reported hypertension among adults aged between 20 and 70 years^19^. One study covering adults aged 20 years and above from Jabalpur city showed that high BMI, waist-circumference and waist-hip ratio was associated with pre-hypertension and hypertension level blood pressure^20^. However, with the higher rates of chronic diseases and differences in adiposity measures among older adults of India, the present study focuses on the 60 years and above males and females who are highly vulnerable.

Owing to the literature, the present study aims to analyze the sex disparity in the relationship of anthropometric indices like BMI, waist circumference and waist-hip ratio with measured Hypertension among Indian older adults. The rationale of such analyses is as follows: First, even though there is ample evidence concerning the association of obesity and Hypertension among Indian adults, most of the findings are not nationally representative. A nationally representative data can provide an idea for other developing nations with the same epidemiological transition level as India. Thus, the present study can be used to reduce the knowledge gap in other developing countries. Second, although we know the prevalence of hypertension differs across sex in various age groups, the sex disparity in the association of anthropometric indices and hypertension is still left unexplored. It would be an exciting finding to explore the wide sex disparity of hypertension across age and the changes in the anthropometric measures among older males and females together affects this association. Although inequality in the prevalence of hypertension or in the association of hypertension with adiposity measures exists for other spheres too like urban–rural, rich–poor, the differences between the male and female sex persist in every age group and may increase with increasing age. Moreover, with the biological differences across the life course can immensely differentiate the association among males and females. For example, reproductive changes in the life of females, changes in lifestyle and alteration in physical work after retirement. Third, there is an incremental disparity between self-reported and diagnosed hypertension prevalence^21^. The prevalence of self-reported disease status in India has often been significantly understated than the actual disease burden^22^. Moreover, the recently concluded, nationally representative, Longitudinal Ageing Study in India (LASI) wave-I report stated that one in every five Indians aged 60 years and above were unaware of hypertension^5^. Utilizing the data from baseline wave of LASI, the present study provides an opportunity to look into the measured hypertension prevalence among older Indian adults. Further, we examined the sex difference in the relationship between obesity-related measures and hypertension among older adults in India.

## Methods

### Data source

The present study used data from the LASI’s baseline wave, conducted during 2017–18. The survey is a joint undertaking of the Harvard T.H. Chan School of Public Health, the International Institute for Population Sciences (IIPS), and the University of Southern California (USC)^5^. This nationally-representative longitudinal survey collected vital information on the physical, social, and cognitive well-being of India’s older adults, and is proposed to be followed up for 25 years. The data of over 72,000 individuals aged 45 and above and their spouses (irrespective of age) were collected from all states and union territories of India. The sample is based on a multistage stratified cluster sample design, including three and four distinct rural and urban area selection stages, respectively. The survey provides scientific insights and facilitates a harmonized design which helps in comparing with parallel international studies. Further, the details of sample design, survey instruments, fieldwork, data collection and processing, and response rates are publicly available in the LASI report available on the website Longitudinal Aging Study in India (LASI)^5^.

We used the data of 15,098 males and 16,366 females aged 60 years and above in India. Further, sample of those with overweight/obesity, high-risk waist circumference and waist-hip ratio may differ from the total sample as every older individual did not give consent for the measurements^5^. All the methods were performed in accordance with the relevant guidelines and regulations.

### Outcome variable

The outcome variable was measured hypertension among older adults. Blood pressure was measured among the respondents after their consent (signed/oral). Omron HEM-7121 blood pressure monitors were used to measure the blood pressure of participants. To obtain accurate results participants were asked to avoid exercise, smoking, consuming alcohol or food within 30 min prior to the measurement and no wounds or swell in the area of measurement^5^. Three blood pressure measures were taken with a gap of 1 min for each in the relax position. The average of the last two readings were considered for the statistical analysis. Hypertension is defined as when an individual had systolic blood pressure of more than equals to 140 mmHg and/or diastolic blood pressure of more than equals to 90 mmHg^23^. Therefore, the variable was coded in binary form i.e., hypertension (no/yes).

All the data were collected by the trained investigators after completing their training on methods and process of study. A detailed instruction is also provided to them regarding taking blood pressure, anthropometric measurements and biological specimen collection.

### Explanatory variables

The explanatory variables for the present study were taken into consideration after an extensive literature review. The main explanatory variables were overweight/obesity condition, high-risk waist circumference and high-risk waist-hip ratio of older adults. These body measurements were taken by the trained professionals under standardized protocols. Participants were asked to stand straight against a wall with bare foot onto the base of stadiometer to measure the height. Weight was measured using weighing scale with the participants wearing light clothes without footwear. BMI was calculated by dividing the weight (in kilograms) by the square of height (in m). The respondents having a BMI of 25 and above were categorized as obese/overweight^24^ coded as “yes”, else “no”.

Participant’s waist and hip circumference was measured using a soft measuring tape called gulik tape in standing position with light clothing^5^. High-risk waist circumference was coded as no and yes. Males and females who have waist circumferences of more than 102 cm and 88 cm respectively were considered as having high-risk waist circumference^25^. Further, the high-risk waist-hip ratio was coded as no and yes. Males and females who have a waist-hip ratio of more than equal to 0.90 and 0.85 respectively were considered as having a high-risk waist-hip ratio^25^.

Further, age was grouped into young old (60–69 years), old–old (70–79 years), and oldest-old (80+ years). Education was coded as no education/primary schooling not completed, primary completed, secondary completed, and higher and above. Marital status was coded as currently married, widowed, and others (separated/never married/divorced). Working status was coded as working, retired, and not working. Tobacco and alcohol consumption was coded as no and yes. Physical activity status was coded as frequent (every day), rare (more than once a week, once a week, one to three times in a month), and never^26^. And the family history of hypertension was coded as “no” or “yes”.

The monthly per capita expenditure (MPCE) quintile was assessed using household consumption data. The details of the measure are published elsewhere^5^. The variable was divided into five quintiles i.e., from poorest to richest. Religion was coded as Hindu, Muslim, Christian, and Others. Caste was recoded as Scheduled Tribes, Scheduled Castes, Other Backward Classes, and others. The Scheduled Castes include a group of the population that is socially segregated and financially/economically by their low status as per Hindu caste hierarchy. The Scheduled Castes and Scheduled Tribes are among the most disadvantaged socioeconomic groups in India. The Other Backward Classes are considered low in the traditional caste hierarchy, but include the intermediate socioeconomic groups. The “others” caste category is identified as those having higher social status. Place of residence was coded as rural and urban. The region was coded as North, Central, East, Northeast, West, and South.

### Statistical analysis

We reported descriptive statistics (percentage) along with the estimates from bivariate analysis. Additionally, we used binary logistic regression analysis^27^ to examine the association between the outcome variable (hypertension) and putative risk or protective factors. The multivariable analysis had four models to explain the adjusted estimates. Model-1 provides the adjusted estimates for the explanatory variables. Model-2, model-3, and model-4 provide the interaction effects^28,29^ of anthropometric measures of obesity and sex on hypertension among older adults. STATA 14 was used to analyze the dataset. Model-1 represents the adjusted odds ratio for all background characteristics; model-2, 3 and 4 were adjusted for all the background characteristics and represent the interaction effects^30^.

An "interaction variable" is a variable that is created from an initial set of variables to either fully or partially describe the interaction that is present. In exploratory statistical analyses, it is typical to employ original variable products as the foundation for assessing the presence of interaction, with the option of later adding other more realistic interaction variables. Multiple interaction variables are constructed when there are more than two explanatory variables, with pairwise-products indicating pairwise interactions and higher order products representing higher order interactions.

Thus, for a response *Y* and two variables *x*_1_ and *x*_2_ an *additive* model would be:

$${\text{Y}} = \upalpha +\upbeta _{{1}} {\text{x}}_{{1}} +\upbeta _{{2}} {\text{x}}_{{2}} +\upvarepsilon _{0}$$

In contrast to this,

$${\text{Y}} = \upalpha +\upbeta _{{1}} {\text{x}}_{{1}} +\upbeta _{{2}} {\text{x}}_{{2}} + (\upbeta _{{3}} {\text{x}}_{{\text{s}}} *{\text{x}}_{{\text{a}}} )\upvarepsilon _{0}$$

where Y is dependent variable (various Hypertension) and α is intercept, x_1_ is individual level independent variable, x_2_ is individual level independent variable, x_a_ is alcohol users, x_s_ is smokers, (β_3_ x_s_ * x_a_ ) is the interaction of sex and obesity related indicators and ε_0_ is error. Models lacking the interaction term d (x_1 *_ x_2_) are frequently presented, although this confuses the main effect and interaction effect (i.e., without specifying the interaction term, it is possible that any main effect found is actually due to an interaction).

### Ethics approval and consent to participate

The data is freely available in the public domain and survey agencies that conducted the field survey for the data and biomarker collection have collected informed consent "oral consent or signed consent" from the respondents. The study was approved by the Health Ministry’s Screening Committee, the Indian Council of Medical Research (ICMR), and the Institutional Review Boards at IIPS and its collaborating institutions. The survey ensured international ethical standards of confidentiality, anonymity, and informed consent.

## Results

Table 1 presents the characteristics of 15,098 males and 16,366 females aged 60 years and above in India. Nearly half of the older participants belonged to the 60–69 age category (58% male and 59% female). About 53% of males and 81% of females were illiterate or not completed primary education. The proportion of currently married males (81%) was higher than that of females (44%). Moreover, 18% and 26% of males and females were found overweight/obesity. High-risk waist circumference was observed among 9% males and 37% females. Furthermore, 75% of males and 78% of females had a high-risk waist-hip ratio. Approximately 44% of overweight/obese older adults had hypertension. About, 47% of males and 43% of females with high-risk waist circumference suffered from hypertension. In contrast, 37% of males and 40% of females with a high waist-hip ratio suffered from hypertension. Hypertension is significantly higher among the oldest-old males and females. Widowed males (37%) and females (41.3%) had a higher chance of suffering from hypertension than their married counterparts. Retired males and females had a higher chance of suffering from hypertension than those currently working. Older males and females who never involved in physical activity suffered from hypertension. Besides, 39% of males and females who lived in urban areas were suffering from hypertension. In terms of region, older adults residing in the northeast region had a higher prevalence of hypertension.

**Table 1:** Sample characteristics and prevalence of hypertension among older males and females in India, 2017–18.

Background characteristics	Male		Female		Hypertensive male	Hypertensive female	p-value
	Sample	%	Sample	%	%	%	
**Obese/overweight**^a^							
No	11,132	82.4	10,719	73.7	31.8	35.5	< 0.001
Yes	2377	17.6	3822	26.3	44.3	44.7	0.080
**High risk waist circumference**^a^							
No	12,303	91.1	9155	63	32.7	34.8	0.898
Yes	1205	8.9	5387	37.1	47.4	43.3	0.007
**High risk waist-hip ratio**^a^							
No	3318	24.6	3123	21.5	24.5	31.3	< 0.001
Yes	10,184	75.4	11,402	78.5	37.1	39.7	0.277
**Age (in years)**							
Young-old	8730	57.8	9678	59.1	33.7	36.9	0.101
Old-old	4702	31.1	4803	29.4	35.4	39.3	< 0.001
Oldest-old	1666	11	1886	11.5	31.1	42.1	< 0.001
**Education**							
No education/primary not completed	8018	53.1	13,314	81.4	32.1	37.4	< 0.001
Primary completed	2235	14.8	1297	7.9	32.5	43.2	< 0.001
Secondary completed	3096	20.5	1297	7.9	36.8	43	0.061
Higher and above	1748	11.6	458	2.8	39.3	32.2	0.153
**Marital status**							
Currently married	12,242	81.1	7211	44.1	33.2	34.3	0.048
Widowed	2489	16.5	8837	54	37	41.3	0.035
Others	366	2.4	318	2	37.3	42.2	0.223
**Working status**							
Working	6613	43.8	3108	19	31.1	35.2	0.321
Retired	7907	52.4	5593	34.2	36.4	40.4	0.061
Not working	578	3.8	7665	46.8	34.3	37.7	0.475
**Tobacco consumption**							
No	6197	41.1	12,706	77.6	35.4	38.4	0.396
Yes	8901	59	3660	22.4	33	37.1	< 0.001
**Alcohol consumption**							
No	10,939	72.5	15,943	97.4	32	38	< 0.001
Yes	4159	27.6	423	2.6	38.9	43.2	0.252
**Physical activity status**							
Frequent	3706	24.6	1966	12	32.2	35.1	0.021
Rare	2360	15.6	1672	10.2	30	33.6	0.145
Never	9031	59.8	12,729	77.8	35.8	39.3	0.010
**Family history of hypertension**							
No	12,151	80.5	12,826	78.4	33.5	37.9	< 0.001
Yes	2947	19.3	3540	21.6	36.1	39.0	0.100
**MPCE quintile**							
Poorest	3145	20.8	3681	22.5	33.4	39.7	0.003
Poorer	3219	21.3	3611	22.1	32.5	38	0.151
Middle	3262	21.6	3331	20.4	33.8	38.6	0.012
Richer	2902	19.2	3136	19.2	35.8	37.1	0.187
Richest	2570	17	2607	15.9	34.4	36.9	0.100
**Religion**							
Hindu	12,386	82	13,484	82.4	33.1	37.3	< 0.001
Muslim	1769	11.7	1781	10.9	34.1	42.1	< 0.001
Christian	388	2.6	511	3.1	40.9	45.3	0.066
Others	555	3.7	590	3.6	46.8	40	0.043
**Caste**							
Scheduled Castes	2836	18.8	3113	19	32.7	37.8	0.077
Scheduled Tribes	1166	7.7	1389	8.5	35.6	38.6	0.429
Other Backward Classes	6925	45.9	7308	44.7	33.4	37.1	0.035
Others	4172	27.6	4556	27.8	35.3	40	< 0.001
**Place of residence**							
Rural	10,879	72.1	11,322	69.2	32	37.4	< 0.001
Urban	4219	28	5044	30.8	39.2	39.9	0.200
**Region**							
North	1863	12.3	2096	12.8	36.5	37.6	0.964
Central	3395	22.5	3202	19.6	27.2	33.7	< 0.001
East	3713	24.6	3729	22.8	32.7	38.3	< 0.001
Northeast	437	2.9	497	3	45	42.4	0.060
West	2457	16.3	2941	18	38.8	39.2	0.168
South	3233	21.4	3900	23.8	36.1	40.6	< 0.001
Total	15,098	100	16,366	100	33.9	38.2	< 0.001

^a^The sample may differ as all older adults did not give consent for the measurements; Difference = Male–Female.

**Table 2:** Logistic regression estimates for hypertension among older adults in India, 2017–18

Background characteristics	Model-1	Model-2	Model-3	Model-4
	AOR 95% CI	AOR 95% CI	AOR 95% CI	AOR 95% CI
**Obese/overweight**				
No	Ref		Ref	Ref
Yes	1.37* (1.27, 1.47)		1.36* (1.26, 1.46)	1.34* (1.25, 1.45)
**High risk waist circumference**				
No	Ref	Ref		Ref
Yes	1.16* (1.08, 1.25)	1.40* (1.31, 1.50)		1.21* (1.11, 1.3)
**High risk waist-hip ratio**				
No	Ref	Ref	Ref	
Yes	1.42* (1.32, 1.51)	1.19* (1.10, 1.28)	1.42* (1.32, 1.51)	
**Age (in years)**				
Young-old	Ref	Ref	Ref	Ref
Old-old	1.16* (1.10, 1.23)	1.16* (1.10, 1.23)	1.16* (1.10, 1.23)	1.16* (1.10, 1.23)
Oldest-old	1.20* (1.10, 1.31)	1.20* (1.10, 1.31)	1.20* (1.10, 1.31)	1.20* (1.10, 1.32)
**Sex**				
Male	Ref			
Female	0.97 (0.9, 1.04)			
**Education**				
No education/primary not completed	Ref	Ref	Ref	Ref
Primary completed	1.09* (1.01, 1.18)	1.09* (1.01, 1.18)	1.09* (1.01, 1.18)	1.09* (1, 1.18)
Secondary completed	1.14* (1.06, 1.24)	1.14* (1.05, 1.23)	1.14* (1.06, 1.23)	1.13* (1.05, 1.23)
Higher and above	1.11* (1, 1.24)	1.10 (0.99, 1.23)	1.11 (1, 1.24)	1.10 (0.99, 1.23)
**Marital status**				
Currently married	Ref	Ref	Ref	Ref
Widowed	1.34* (1.26, 1.42)	1.34* (1.26, 1.42)	1.34* (1.26, 1.42)	1.34* (1.26, 1.42)
Others	1.21* (1.03, 1.41)	1.21* (1.04, 1.42)	1.21* (1.04, 1.41)	1.21* (1.03, 1.41)
**Working status**				
Working	Ref	Ref	Ref	Ref
Retired	1.09* (1.02, 1.17)	1.09* (1.02, 1.17)	1.09* (1.02, 1.17)	1.09* (1.02, 1.16)
Not working	1.05 (0.97, 1.14)	1.06 (0.98, 1.15)	1.06 (0.98, 1.15)	1.06 (0.98, 1.15)
**Tobacco consumption**				
No	Ref	Ref	Ref	Ref
Yes	0.96 (0.9, 1.01)	0.96 (0.90, 1.02)	0.96 (0.9, 1.01)	0.96 (0.9, 1.01)
**Alcohol consumption**				
No	Ref	Ref	Ref	Ref
Yes	1.23* (1.15, 1.33)	1.24* (1.15, 1.33)	1.23* (1.15, 1.33)	1.24* (1.15, 1.33)
**Physical activity**				
Frequent	Ref	Ref	Ref	Ref
Rare	0.97 (0.89, 1.06)	0.97 (0.89, 1.06)	0.97 (0.89, 1.06)	0.97 (0.89, 1.06)
Never	1.01 (0.94, 1.09)	1.01 (0.94, 1.09)	1.01 (0.94, 1.09)	1.01 (0.94, 1.09)
**Family history of hypertension**				
No	Ref	Ref	Ref	Ref
Yes	1.05 (0.99, 1.12)	1.05 (0.99, 1.12)	1.05 (0.99, 1.12)	1.05 (0.99, 1.12)
**MPCE quintile**				
Poorest	Ref	Ref	Ref	Ref
Poorer	0.95 (0.88, 1.02)	0.95 (0.88, 1.02)	0.95 (0.88, 1.03)	0.95 (0.88, 1.03)
Middle	1.00 (0.92, 1.08)	1.00 (0.92, 1.08)	1.00 (0.92, 1.08)	1.00 (0.92, 1.08)
Richer	0.93 (0.86, 1.01)	0.93 (0.86, 1.01)	0.93 (0.86, 1.01)	0.93 (0.86, 1.01)
Richest	0.86* (0.79, 0.94)	0.86* (0.79, 0.94)	0.86* (0.79, 0.94)	0.86* (0.79, 0.94)
**Religion**				
Hindu	Ref	Ref	Ref	Ref
Muslim	1.22* (1.13, 1.32)	1.22* (1.13, 1.32)	1.22* (1.13, 1.32)	1.22* (1.13, 1.32)
Christian	1.07 (0.97, 1.18)	1.07 (0.97, 1.18)	1.07 (0.97, 1.18)	1.07 (0.97, 1.19)
Others	1.24* (1.11, 1.4)	1.24* (1.11, 1.4)	1.24* (1.11, 1.4)	1.24* (1.11, 1.4)
**Caste**				
Scheduled Castes	Ref	Ref	Ref	Ref
Scheduled Tribes	1.30* (1.18, 1.43)	1.30* (1.18, 1.43)	1.30* (1.18, 1.43)	1.31* (1.19, 1.44)
Other Backward Classes	0.91* (0.84, 0.98)	0.91* (0.85, 0.98)	0.91* (0.84, 0.98)	0.91* (0.84, 0.98)
Others	1.01 (0.93, 1.09)	1.01 (0.93, 1.09)	1.01 (0.93, 1.09)	1.00 (0.93, 1.09)
**Place of residence**				
Rural	Ref	Ref	Ref	Ref
Urban	0.99 (0.93, 1.05)	0.99 (0.93, 1.05)	0.99 (0.93, 1.05)	0.99 (0.93, 1.05)
**Region**				
North	Ref	Ref	Ref	Ref
Central	0.89* (0.81, 0.98)	0.89* (0.81, 0.98)	0.89* (0.81, 0.98)	0.89* (0.81, 0.98)
East	1.06 (0.98, 1.16)	1.06 (0.97, 1.15)	1.06 (0.97, 1.15)	1.07 (0.98, 1.16)
Northeast	1.17* (1.05, 1.3)	1.17* (1.05, 1.3)	1.17* (1.05, 1.3)	1.17* (1.05, 1.31)
West	1.04 (0.95, 1.14)	1.04 (0.95, 1.14)	1.04 (0.95, 1.14)	1.03 (0.94, 1.13)
South	1.25* (1.15, 1.36)	1.25* (1.15, 1.36)	1.25* (1.15, 1.36)	1.24* (1.14, 1.35)
**Obese/overweight # sex**				
Yes # male		Ref		
No # male		0.67* (0.61, 0.74)		
No # female		0.67* (0.61, 0.74)		
Yes # female		0.84* (0.75, 0.95)		
**High risk waist circumferences # sex**				
Yes # male			Ref	
No # male			0.80* (0.70, 0.91)	
No # female			0.78* (0.68, 0.9)	
Yes # female			0.89* (0.78, 0.99)	
**High risk waist-Hip ratio # sex**				
Yes # male				Ref
No # male				0.60* (0.55, 0.66)
No # female				0.76* (0.68, 0.84)
Yes # female				0.90* (0.83, 0.97)

Ref: Reference; #: Interaction; *if p < 0.05; AOR: Adjusted Odds Ratio; Model-1 represents the adjusted odds ratio adjusted for all background characteristics; model-2, 3 and 4 were adjusted for all the background characteristics and represents the interaction effects.

Table 2 presents the multivariable association of hypertension with the explanatory variables among older adults in India. Model 1 shows that the odds of hypertension were 1.37 times (CI: 1.27–1.47), significantly higher among older adults who were obese or overweight compared to those with no overweight/obese condition. Older adults with high-risk waist circumference and waist-hip ratio had 1.16 times (CI: 1.08–1.25) and 1.42 times (CI: 1.32–1.51) higher odds of suffering from hypertension, respectively compared to their counterparts with no high-risk waist circumference or waist-hip ratio. The odds of hypertension were 1.20 times (CI: 1.10–1.31) higher among oldest-old than those who belonged to the young-old age category; 1.31 times (CI: 1.26–1.42) higher among widowed than those who were currently married; 1.14 times (CI: 1.06–1.24) higher among older adults who completed secondary education compared to those who were illiterate. Alcohol consumption among older adults was associated with 1.23 times (CI: 1.15–1.33) higher odds of suffering from hypertension. Belonging to households with richest MPCE and Other Backward Caste (OBC) was advantageous for older adults in reducing hypertension risk. Further, individuals belonging to the southern region had 1.25 times (CI: 1.15–1.36) higher odds of suffering from hypertension than those who belonged to the northern region.

Model 2, 3, and 4 estimate the adjusted odds ratio after introducing interaction effects. Females who were overweight or obese had lower odds of suffering from hypertension than their overweight/obese male counterparts [OR: 0.84; CI: 0.61–0.74]. Similarly, females who had high-risk waist circumference and waist-hip ratio had lower odds of suffering from hypertension compared to their male counterparts with the similar anthropometric status.

## Discussion

Recent studies have observed that hypertension prevalence rates are on the rise in developing countries compared to developed countries with no improvement in awareness or control rates^31–33^. Based on an extensive nationally representative data of the older population in India, we examined BMI, waist-to-hip ratio and waist circumference to assess obesity and investigate their associations with hypertension. The overall 33.9% and 38.2% prevalence of hypertension among older males and females, respectively, indicates that the condition affects a sizable proportion of older Indian adults. The current prevalence estimates are comparable and higher than the pooled estimates among the Indian adults (18 years and above), with 29.8% being hypertensive^8^. However, in a recent study among multi-ethnic Asian populations, 33.1% Malaysian adults and 31.5% Chinese adults were hypertensive^34^. Another cross-sectional study in Indonesia found 31.0% of males and 35.4% of females having hypertension^35^.

Consistent with past studies, hypertension among the study sample was associated with their BMI, waist-to-hip ratio and waist circumference^36–40^. Several population-based studies have compared such anthropometric indicators with incident hypertension, and there are inconsistencies in the findings^41–43^. Some studies have specifically shown waist circumference to have an independent effect on the risk of hypertension and claimed that it could replace waist-to-hip ratio and BMI as a simple indicator for weight management^44–46^. A large community-based cross-sectional study in India reported the prevalence of isolated systolic hypertension as 2.8% among patients with normal BMI and 21.1% among those with a higher BMI^47^. Similarly, multiple studies have shown an increased prevalence of hypertension in people with adverse obesity-related measures^48–50^.

The prevalence of hypertension increased with age in males and females, also observed in the previous population-based studies^51–53^. The current findings further showed that older adults retired from work had increased odds of hypertension compared to their currently working counterparts. For aged people, especially the “oldest-old”, the “healthy worker effect” (survival bias) may exist^54^, where the prevalence of cardiovascular diseases might have caused retirement of individuals, thus leading to the inverse causality in the observed association. Although the regression results showed no statistical significance, the urban–rural gradient observed in the bivariate analysis is similar to previous findings from India that showed that the prevalence is higher in urban areas among males and females^8,55^. This urban–rural differential has reversed in many high- income countries where hypertension is more in the rural populations^56^. Thus, the current result shows that the changes influencing the reversal of concentration of hypertensive people in urban areas have not yet happened in India. However, the finding of a recent study suggested an urban–rural convergence of hypertension in India that is taking place due to a rapid urbanization with consequent changes in lifestyles and the resultant increase in overweight and obesity^57^.

Further, the significant positive association of alcohol consumption with prevalence of hypertension among older adults are consistent with several studies in different parts of the world that demonstrated the negative effects of higher levels of frequent drinking on cardiovascular health^58–60^. A recent study also established the causal pathway between moderate-to-heavy alcohol drinking and prevalence of hypertension^61^. The current study also found a higher percent of older males reporting tobacco use than older females which is parallel with other studies in India showing higher rates of tobacco use in males and those from lower socioeconomic groups^62,63^, however, the multivariate analysis showed no significant association of tobacco use with hypertension. Future studies may focus on this aspect. Also, the finding that showed a higher prevalence of hypertension among people who are disadvantaged in many of the socioeconomic indicators such as household per capita expenditure and marital status was in accordance with previous studies in India^64^. This reflects the changes in morbidity dynamics in Indian context since the burden of chronic diseases including hypertension begins to shift from higher to lower socioeconomic groups in the later stages of the epidemiological transition^65,66^.

Another important finding of the present study was the significant association of Muslim and other religious groups with higher prevalence of hypertension. The finding is in concordance with prior research showing the protective role of religion and religious behaviors on high blood pressure^67,68^. This suggests the need for a comprehensive evaluation of the role of specific religions in hypertension control. The analysis of regional variations in the prevalence rates also concurs with earlier studies that have shown a high prevalence of hypertension in South India^7,69,70^. The high prevalence of hypertension in South India maybe attributed to the higher presence of cardio-metabolic risk factors such as high BMI and central obesity in this region^8,71^.

It is reported that frequently used anthropometric indices in epidemiological studies such as BMI, waist circumference and waist-hip ratio, measure body fat with different results across sex and in different populations due to genetic and lifestyle differences^72^, which makes a comparison between studies difficult. The increased high-risk waist-circumference among older females than males in the current study is also in line with previous research reporting an accelerated shift toward a more central fat distribution in females after menopause while males have a centrally located fat distribution during adulthood^73,74^, resulting in increased risk of hypertension in old age females. While we have shown the prevalence of hypertension across various factors such as obesity-related-measures, alcohol consumption and physical inactivity that were significantly different among older males and females, results suggested that sex is a strong confounder.

The analyses of interactive effects of sex on the observed associations were conducted in order to intuitively show and compare the relationships between obesity measures and hypertension among older males and females. Findings of several clinical and epidemiological studies showing higher rates of hypertension with aging in females versus males, suggest that sex and/or sex hormones have a prominent role in hypertension^75–77^. Such studies showed that sex differences exist in hypertension and post-menopausal females have pronounced increases in hypertension compared to males in the same age groups^78–80^. The current findings in line with previous studies observed the effect of sex on the association of obesity related measures and hypertension suggesting that there are physiological differences among older males and females regarding the distribution of body fat and related cardiovascular health outcomes^81–84^. However, whether the sex differential is due to biological factors, inadequate treatment due to physician inertia or adherence, or inappropriate drug choices or sex-related differences in medications, all has to be further investigated.

The strengths of this study include the nationally representative survey with a large sample size. Also, various covariates consisting of separate obesity-related anthropometric measures increase the validity of the study. Nevertheless, cross-sectional design of the study is a major limitation that provides only a one-time measurement of obesity and related indices and hypertension. Hence, it is unfeasible to state any causal inferences from the study. Further, as health literacy, self-monitoring and taking medication would influence blood pressure significantly, not including such variables in the current analysis might lead to misclassification bias, which need to be addressed in future studies with longitudinal design.

## Conclusion

All three indicators related to obesity studied here have shown significant association with the prevalence of hypertension suggesting the importance of weight reduction in prevention of hypertension. The findings also suggested the larger magnitude of the association between obesity, high-risk waist circumference, high-risk waist-hip ratio and hypertension among older males than females. The study also highlights the importance of measuring obesity and central adiposity in older individuals and using such measures as screening tool for timely identification of hypertension. Since it carries a higher risk for all-cause, as well as cardiovascular mortality, among modifiable risk factors, hypertension management should be a key health care priority for older population and policies should be developed with a focus on sex differences in related risks.

## Data Availability

The study uses secondary data which is available on reasonable request through https://www.iipsindia.ac.in/content/lasi-wave-i. The data used in the study can be deposited publicly. The data will also be available on request from the corresponding author.
